# Association of a simple SACAF score with bystander witnessed sudden death due to ventricular tachyarrhythmias in a multicenter cohort

**DOI:** 10.1038/s41598-021-00940-0

**Published:** 2021-11-04

**Authors:** Mei-Yao Wu, Ming-Shien Wen, Mien-Cheng Chen, Chia-Ti Tsai, Tsu-Juey Wu, Wei-Chieh Lee, Yen-Nien Lin, Shih-Sheng Chang, Kuan-Cheng Chang

**Affiliations:** 1 School of Post-Baccalaureate Chinese Medicine, China Medical University, Taichung, Taiwan; 2 Department of Chinese Medicine, China Medical University Hospital, Taichung, Taiwan; 3 Division of Cardiology, Chang Gung Memorial Hospital, Linkou, Taoyuan, Taiwan; 4 College of Medicine, Chang Gung University, Taoyuan, Taiwan; 5 Division of Cardiology, Department of Internal Medicine, Kaohsiung Chang Gung Memorial Hospital, Kaohsiung, Taiwan; 6 Division of Cardiology, Department of Internal Medicine and Cardiovascular Center, National Taiwan University Hospital, 7, Chung-Shan South Road, Taipei, 10002 Taiwan; 7 Graduate Institute of Clinical Medicine, College of Medicine, National Taiwan University, Taipei, Taiwan; 8 Cardiovascular Center and Department of Internal Medicine, Taichung Veterans General Hospital, Taichung, Taiwan; 9 Institute of Clinical Medicine, National Yang Ming Chiao Tung University, Taipei, Taiwan; 10 Institute of Clinical Medicine, College of Medicine, National Cheng Kung University, Tainan, Taiwan; 11 Division of Cardiovascular Medicine, Department of Internal Medicine, China Medical University Hospital, 2, Yude Road, Taichung, 40447 Taiwan; 12 Cardiovascular Research Laboratory, China Medical University Hospital, Taichung, Taiwan; 13 School of Medicine, China Medical University, Taichung, Taiwan; 14 Graduate Institute of Biomedical Sciences, China Medical University, Taichung, Taiwan

**Keywords:** Cardiology, Risk factors

## Abstract

Out-of-hospital cardiac arrest (OHCA) remains a major threat to public health worldwide. OHCA patients presenting initial shockable ventricular tachycardia/ventricular fibrillation (VT/VF) rhythm have a better survival rate. We sought to develop a simple SACAF score to discriminate VT/VF from non-VT/VF OHCAs based on the Taiwan multicenter hospital-based registry database. We analyzed the in- and pre-hospital data, including demographics, baseline comorbidities, response times, automated external defibrillator information, and the 12-lead ECG recording closest to the OHCA event in bystander-witnessed OHCA patients. Among the 461 study patients, male sex (OR 2.54, 95% CI = 1.32–4.88, *P* = 0.005), age ≤ 65 years (OR 2.78, 95% CI = 1.64–4.70, *P* < 0.001), cardiovascular diseases (OR 2.97, 95% CI = 1.73–5.11, *P* < 0.001), and atrial fibrillation (AF) (OR 2.36, 95% CI = 1.17–4.76, *P* = 0.017) were independent risk factors for VT/VF OHCA (n = 81) compared with non-VT/VF OHCA (n = 380). A composite SACAF score was developed (male Sex, Age ≤ 65 years, Cardiovascular diseases, and AF) and compared with the performance of a modified CHA_2_DS_2_-VASc score (Cardiovascular diseases, Hypertension, Age ≥ 75 years, Diabetes, previous Stroke, Vascular disease, Age 65–74 years, female Sex category). The area under the receiver operating characteristic curve (AUC) of the SACAF was 0.739 (95% CI = 0.681–0.797, *P* < 0.001), whereas the AUC of the modified CHA_2_DS_2_-VASc was 0.474 (95% CI = 0.408–0.541, *P* = 0.464). A SACAF score of ≥ 2 was useful in discriminating VT/VF from non-VT/VF OHCAs with a sensitivity of 0.75 and a specificity of 0.60. In conclusion, the simple SACAF score appears to be useful in discriminating VT/VF from non-VT/VF bystander-witnessed OHCAs and the findings may also shed light on future mechanistic evaluation.

## Introduction

Out-of-hospital cardiac arrest (OHCA), accounting for up to 50% of all cardiovascular deaths, remains a major threat to public health worldwide^1^. The incidence of emergency medical services (EMS)-treated OHCA ranges from 30.0 to 97.1 individuals per 100,000 in the general population^2^. In general, the survival to hospital discharge rates of OHCA patients ranges between 3.1 and 20.4%^2^. Among the OHCA patients who are bystanders witnessed with initial shockable rhythm, ventricular tachycardia/ventricular fibrillation (VT/VF), the survival rate (11.7–47.4%) was higher compared with those who did not present VT/VF rhythm^2^.

Given the better prognosis of OHCA patients with an initial VT/VF rhythm, it is imperative to identify the risk factors for VT/VF OHCA to develop effective prevention strategies to improve survival for these patients. The incidence of initial VT/VF in general OHCA patients varies from continent to continent, with a 20–30% incidence rate in white patients^3–5^ and a 5–10% incidence rate in East Asian populations according to nationwide registries or community-based studies^6–8^. In witnessed nontraumatic OHCA populations, the difference in VT/VF incidence between Western (60–80%) and Eastern (20%) people was even more prominent^9–11^. Although there were significant variations in the incidence of VT/VF OHCA between Eastern and Western patients, both populations did share some common risk factors for VT/VF OHCA. Our previous study of the OHCA registry in a metropolitan city in central Taiwan (THUNDER) showed that male sex, age younger than 65 years old, public location of arrest, and witnessed status were independent predictors of VT/VF OHCA^12^. In a prospective cohort study of OHCA in adults from 10 North American communities, Weisfeldt et al. showed that younger age and male sex were independently associated with initial VT/VF in both bystander-witnessed arrests and EMS-witnessed arrests^11^.

In addition to demographic factors, ECG, a simple and convenient noninvasive tool, has been widely used to risk-stratify or predict subsequent VT/VF occurrence in general populations or in patients with cardiovascular diseases. ECG of atrial fibrillation (AF) rhythm has been shown to have a higher risk of VT/VF sudden cardiac death (SCD) than non-AF subjects in a nationwide cohort study^13^. In a community-based case–control study, AF was associated with a threefold increased risk of VF OHCA^14^. Apart from the rhythm, several key ECG parameters, including faster resting heart rate, prolongation of the QRS, QTc and JTc intervals, delayed intrinsicoid deflection, wider QRS-T angle, and longer T wave peak to T wave end (TpTe) measured by 12-lead ECG, have been identified as independent risk factors for SCD in general populations or in patients with coronary artery disease^15–20^. In the acute phase of ST-elevation myocardial infarction, it has been shown that QTc_PVC_ interval, TpTe interval, TpTe/QT ratio, and TpTe/QT_PVC_ ratio were independent risk factors in predicting VT/VF^21^. However, whether these ECG predictors of VT/VF can also be applied to Asian people remains inconclusive.

In the context of a multifactorial milieu, incorporation of multiple risk factors into a scoring system to predict VT/VF appears to be a reasonable approach. Recently, Nof et al. demonstrated that a high CHA_2_DS_2_-VASc score was inversely associated with VT/VF events in patients enrolled in the Multicenter Automatic Defibrillator Implantation Trial with Cardiac Resynchronization Therapy (MADIT-CRT)^22^. With this background information, we were motivated to conduct a multicenter study to identify the risk factors for initial VT/VF by analyzing the demographics, comorbidities, and ECG parameters in witnessed OHCA patients from 5 tertiary-care medical centers in Taiwan. With this approach, we sought to develop a useful scoring tool to effectively predict VT/VF OHCA. The ultimate goal was to better understand the regional risk factors for VT/VF OHCA and to pave the way for implementing evidence-based preventive management for VT/VF OHCA patients in this region.

## Methods

### Study design and setting

The Taiwan Multicenter Hospital-based Registry Targeting Sudden Unexpected Death (THREATEN) study consists of five medical centers spanning North and South Taiwan, including National Taiwan University Hospital (NTUH), Chang Gung Memorial Hospital (Linkou, CGMH-LK), Taichung Veteran General Hospital (VGHTC), China Medical University Hospital (CMUH), and Chang Gung Memorial Hospital (Kaohsiung, CGMH-KH). The study protocol was reviewed and approved by the Research Ethics Committee of each center with the joint analysis center at CMUH (CMUH107-REC1-092). All study procedures were performed in accordance with relevant guidelines and regulations. The Research Ethics Committee waived the requirement for obtaining a signed consent form from all subjects because of the retrospective database research design of the present study.

Nontraumatic bystander-witnessed OHCA in patients older than 20 years old occurring between January 1, 2013, and December 31, 2019, were included in this study. Patients on whom automated external defibrillators (AEDs) were not used based on EMS records, who did not have complete demographic data, or who did not have 12-lead ECG records in the hospital before the OHCA event were excluded. Demographic information, EMS records, and hospital data according to the Utstein Style were included in the EMS run sheets of patients with OHCA transported by 119 EMS^23, 24^. The data included age, sex, witnessed status, first documented cardiac rhythm, location of arrest, time course of resuscitation, cardiopulmonary resuscitation (CPR), AED use, return of spontaneous circulation (ROSC), survival, and complete 12-lead ECG before the OHCA event.

### Data collection

**Figure 1:**
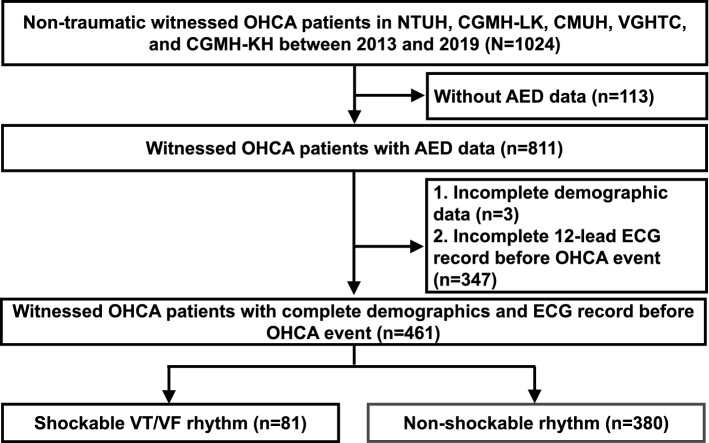
Flow diagram depicting the enrollment of witnessed out-of-hospital cardiac arrest (OHCA) patients. Of the 1024 witnessed OHCA patients in NTUH, CGMH-LK, CMUH, VGHTC, and CGMH-KH between January 1, 2013, and December 31, 2019, 811 patients had complete AED data. After excluding patients with incomplete demographic data (n = 3) or no 12-lead ECG records before OHCA events (n = 347), a total of 461 patients were included in the analysis. There were 81 patients with initial shockable VT/VF rhythm and 380 patients with non-shockable rhythm. AED, Automated External Defibrillator; CGMH-KH, Kaohsiung Chang Gung Memorial Hospital; CGMH-LK, Chang-Gung Memorial Hospital at Lin-Ko; CMUH, China Medical University Hospital; NTUH, National Taiwan University Hospital; VGHTC, Taichung Veterans General Hospital; VT, ventricular tachycardia; VF, ventricular fibrillation.

The electronic medical records before the OHCA event of the enrolled nontraumatic bystander-witnessed OHCA patients in these five hospitals were reviewed. Patients without complete demographic and 12-lead ECG data before the OHCA event were excluded (Fig. 1). Baseline comorbidities, including diabetes mellitus, hypertension, hyperlipidemia, cardiovascular diseases (including coronary heart diseases, arrhythmia, heart failure, and peripheral arterial occlusive disease), cerebral vascular accidents, end-stage renal disease, chronic obstructive pulmonary disease, asthma, and cancer, before the OHCA event of the enrolled patients were identified. The 12-lead ECG records closest to the OHCA event of the enrolled patients in these five hospitals were reviewed by cardiologists, and the parameters were extracted. The included patients were separated into two groups according to the initial shockable or non-shockable rhythm.

### Twelve-lead ECG analysis

A standard 12‐lead ECG tracing at 25 mm/s paper speed and 10 mm/mV amplitude prior to and closest to the OHCA event of the enrolled patients was reviewed. Board-certificated cardiologists reviewed ECGs to identify heart rhythm, which included sinus rhythm, AF, and AFL, and bundle branch block or intraventricular conduction delay. ECG data available for evaluation from the commercial algorithm (data management software by MUSE, GE Healthcare, US or Philips PageWriter TC50 Cardiograph, Philips, Netherlands) included ventricular rate, QRS duration, and QTc interval. QTc indicates the correction of the QT interval by heart rate according to Bazett’s formula. ECG left ventricular hypertrophy (LVH) was defined according to the Sokolow-Lyon criteria (RV1 + SV5,6 ≥ 35 mm)^20, 25^. The frontal QRS‐T angle was calculated as the absolute difference in value between the frontal plane QRS axis and the T‐wave axis, which were both available from the commercial algorithm. TpTe was calculated by the tangent method in maximum Tpe among precordial ECG leads^26^.

### Identification of independent predictors to construct the prediction model

Multivariate logistic regression analysis was used to identify the independent risk factors for shockable VT/VF rhythm in witnessed OHCA patients. Variables with a *P* < 0.05 on univariate analyses were considered to represent explanatory variables and were included in the multivariate analysis to identify the independent predictors of initial VT/VF rhythm. Sex, age, cardiovascular disease, ECG parameters of ventricular rate, sinus rhythm, AF, QRS duration, and frontal QRS-T angle were variables entered into a backward elimination logistic regression model. Backward elimination of variables was set to a significance level of 0.05 and based on the probability of the likelihood-ratio statistic and the maximum partial likelihood estimates.

Independent risk factors identified by multivariate logistic regression were used to develop a prediction model for discriminating VT/VF from non-VT/VF OHCAs. The performance of the model in predicting VT/VF rhythm was assessed by analyzing the area under the receiver operating characteristic curve (AUC) as well as the sensitivity and specificity. For comparison, the AUC of modified CHA_2_DS_2_-VASc (Cardiovascular diseases, Hypertension, Age ≥ 75 years, Diabetes, previous Stroke, Vascular disease, Age 65–74 years, female Sex category, with doubled risk weight for stroke and age ≥ 75 years) was also calculated to evaluate whether it was inversely associated with VT/VF events in witnessed OHCA patients.

### Statistical analysis

Continuous data are presented as the mean ± SD, and categorical data are presented as numbers (percentages). For continuous variables in the demographic data and ECG parameters, the differences between the two groups were analyzed by Student’s *t* test. For comparing the categorical variables between the two groups, the Pearson *X*^2^ test or Fisher’s exact test was used.

All statistical analyses were performed using IBM SPSS Statistics version 22 (IBM, Armonk, NY, USA), and a *P* value of < 0.05 was considered statistically significant.

## Results

Of the 1024 witnessed OHCA patients in NTUH, CGMH-LK, CMUH, VGHTC, and CGMH-KH between January 1, 2013, and December 31, 2019, 811 patients had complete AED data (Fig. 1). After excluding patients with incomplete demographic data (n = 3) or no 12-lead ECG records before OHCA events (n = 347), a total of 461 patients were included in the analysis. There were 81 patients with initial shockable VT/VF rhythm and 380 patients with non-shockable rhythm.

Table 1 shows the demographic data of the enrolled witnessed OHCA patients. In VT/VF group, the percentage of males was higher (84.0% vs. 63.2%, *P* < 0.001) and the age was younger (64.0 ± 15.7 vs. 72.1 ± 15.1, *P* < 0.001), compared with the non-VT/VF group. In VT/VF group, the prevalence of cardiovascular diseases (67.9% vs. 41.1%, *P* < 0.001), history of percutaneous coronary intervention (32.1% vs. 10.5%, *P* < 0.001), and smoking habits was higher (34.6% vs. 22.6%, *P* = 0.033) than those in the non-VT/VF group. The baseline comorbidities including diabetes, hypertension, hyperlipidemia, cerebral vascular accidents, end-stage renal disease, chronic obstructive pulmonary disease, asthma, and cancer were similar between the two groups. There was no significant difference in the EMS response time between the two groups (5.8 ± 2.7 min vs. 5.9 ± 2.0 min, *P* = 0.818).

**Table 1:** Demographic data of witnessed OHCA patients.

	VT/VF (n = 81)	Non-VT/VF (n = 380)	*P* value
Sex			**< *0.001***
Male	68 (84.0%)	240 (63.2%)	
Female	13 (16.0%)	140 (36.8%)	
Age, mean	64.0 ± 15.7	72.1 ± 15.1	**< *0.001***
Age group (years old)			**< *0.001***
≤ 65 years old	44 (54.3%)	123 (32.4%)	
> 65 years old	37 (45.7%)	157 (68.6%)	
Smoking	28 (34.6%)	86 (22.6%)	***0.033***
Diabetes mellitus	31 (38.3%)	169 (44.5%)	0.325
Hypertension	58 (71.6%)	244 (64.2%)	0.247
Hyperlipidemia	24 (29.6%)	73 (19.2%)	0.050
Cardiovascular disease	55 (67.9%)	156 (41.1%)	**< *0.001***
Cerebral vascular accidents	14 (17.3%)	103 (27.1%)	0.069
End-stage renal disease	15 (18.5%)	74 (19.5%)	1.000
Chronic obstructive pulmonary disease	5 (6.2%)	43 (11.3%)	0.228
Asthma	4 (4.9%)	27 (7.1%)	0.628
Cancer	13 (16.0%)	97 (25.5%)	0.084
Percutaneous coronary intervention	26 (32.1%)	40 (10.5%)	**< *0.001***
Response time	5.8 ± 2.7	5.9 ± 2.0	0.818

Statistically significant (*P* < 0.05) are given in bold italic.

Data are given as the mean ± SD or n (%). Continuous data were analyzed by Student’s t test, and categorical data were analyzed by the chi-square test.

OHCA, Out-of-hospital cardiac arrest; VT, ventricular tachycardia; VF, ventricular fibrillation.

Table 2 summarizes the ECG parameters closest to the OHCA event of the enrolled witnessed OHCA patients. The median time (interquartile range, IQR) of the 12-lead ECG record closest to the OHCA event was 6.7 months (1.7–24.2 months). In VT/VF group, the ventricular rate was slower (83.3 ± 22.2 vs. 90.0 ± 21.6 bpm, *P* = 0.011) with a lower percentage of sinus rhythm (76.5% vs. 86.6%, *P* = 0.027) but a higher percentage of AF (21.0% vs. 10.3%, *P* = 0.013) compared with non-VT/VF group. Patients presenting VT/VF OHCA exhibited a longer QRS duration (101.5 ± 19.3 ms vs. 93.2 ± 32.6 ms, *P* = 0.028) and a wider frontal QRS-T angle (74.4 ± 61.3° vs. 58.0 ± 55.7°, *P* = 0.018) on a 12-lead ECG before the OHCA event than those presenting non-VT/VF OHCA. There were no significant differences in the percentage of bundle branch block, any degree of atrioventricular block, LVH, or duration of QTc interval and TpTe.

**Table 2:** ECG parameters closest the OHCA event of OHCA patients.

	VT/VF (n = 81)	Non-VT/VF (n = 380)	*P* value
Ventricular rate, mean (/min)	83.3 ± 22.2	90.0 ± 21.6	***0.011***
Sinus rhythm	62 (76.5%)	329 (86.6%)	***0.027***
Atrial fibrillation (AF)	17 (21.0%)	39 (10.3%)	***0.013***
QRS duration (ms)	101.5 ± 19.3	93.2 ± 32.6	***0.028***
Bundle branch block	6 (7.4%)	36 (9.5%)	0.674
Any degree of atrioventricular block	4 (4.9%)	24 (6.6%)	0.801
QTc interval (ms)	457.9 ± 39.8	453.8 ± 50.7	0.492
Left ventricular hypertrophy	10 (12.3%)	35 (9.2%)	0.409
T wave peak to T wave end (ms)	95.2 ± 35.0	97.8 ± 38.4	0.578
Frontal QRS-T angle, mean (°)	74.4 ± 61.3	58.0 ± 55.7	***0.018***

Statistically significant (*P* < 0.05) are given in bold italic.

Data are given as the mean ± SD or n (%). Continuous data were analyzed by Student’s t test, and categorical data were analyzed by the chi-square test.

OHCA, Out-of-hospital cardiac arrest; VT, ventricular tachycardia; VF, ventricular fibrillation.

**Figure 2:**
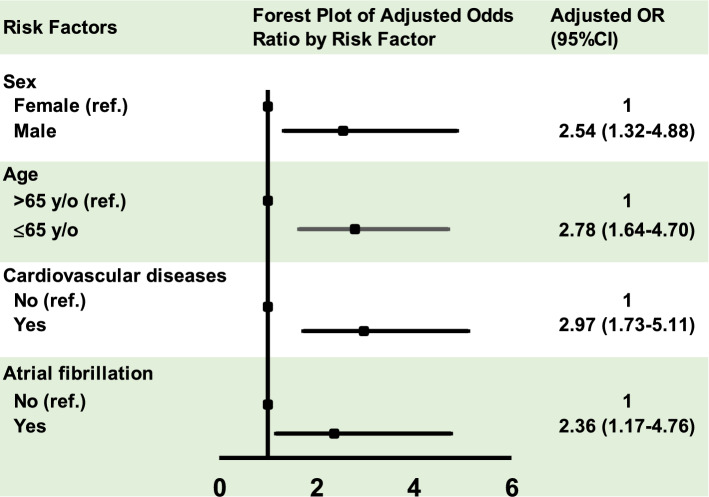
Independent predictors of VT/VF rhythm in witnessed OHCA patients. Males, age ≤ 65 years old, a past history of cardiovascular diseases, and ECG of AF rhythm closest to the OHCA event were independent risk factors for witnessed VT/VF OHCAs identified by using multivariate logistic regression analysis.

Using multivariate logistic regression analysis, we identified that males (OR 2.54, 95% CI 1.32–4.88, *P* = 0.005), age ≤ 65 years old (OR 2.78, 95% CI 1.64–4.70, *P* < 0.001), a past history of cardiovascular diseases (OR 2.97, 95% CI 1.73–5.11, *P* < 0.001), and ECG of AF rhythm closest to the OHCA event (OR 2.36, 95% CI 1.17–4.76, *P* = 0.017) were independent risk factors for witnessed VT/VF OHCAs (Table 3, Fig. 2).

**Table 3:** Multivariate logistic regression: independent predictors for VT/VF in witnessed OHCA patients.

Variables	Univariate analysis		Multivariate analysis	
	OR (95% CI)	*P* value	OR (95% CI)	*P* value
**Sex**				
Female	1.00		–	–
Male	3.05 (1.63–5.72)	***0.001***	2.54 (1.32–4.88)	***0.005***
**Age**				
> 65 years old	1.00		–	–
≤ 65 years old	2.49 (1.53–4.04)	**< *0.001***	2.78 (1.64–4.70)	**< *0.001***
**Cardiovascular diseases**				
No	1.00		–	–
Yes	3.04 (1.83–5.06)	**< *0.001***	2.97 (1.73–5.11)	**< *0.001***
**Atrial fibrillation (AF)**				
No	1.00		–	–
Yes	2.32 (1.24–4.36)	***0.009***	2.36 (1.17−4.76)	***0.017***

Statistically significant (*P* < 0.05) are given in bold italic.

Variables entered on step 1: sex, age, cardiovascular disease, ECG parameters of ventricular rate, sinus rhythm, atrial fibrillation, QRS duration, and frontal QRS-T angle.

OHCA, Out-of-hospital cardiac arrest; VT, ventricular tachycardia; VF, ventricular fibrillation.

**Figure 3:**
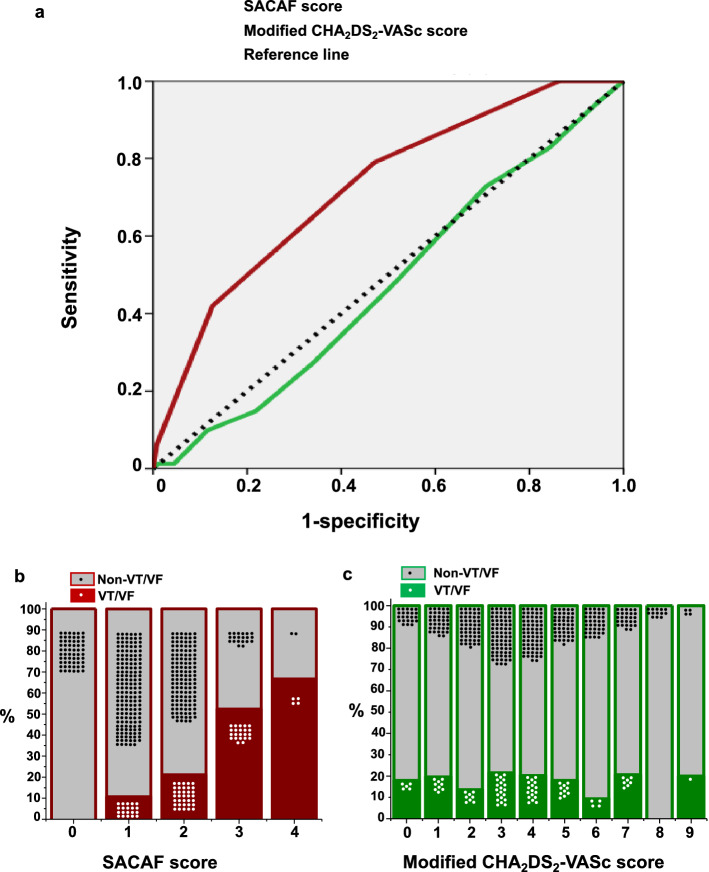
Receiver operating characteristic (ROC) curves of the SACAF score and the modified CHA_2_DS_2_-VASc score for predicting VT/VF rhythm in witnessed OHCA patients. (**A**) ROC curves of the SACAF score (male Sex, Age ≤ 65 years, Cardiovascular diseases, and AF, red line) and the modified CHA_2_DS_2_-VASc score (Cardiovascular diseases, Hypertension, Age ≥ 75 years, Diabetes, previous Stroke, Vascular disease, Age 65–74 years, female Sex category with doubled risk weight for stroke and age ≥ 75 years, green line) were constructed to discriminate VT/VF from non-VT/VF OHCA using the area under the ROC curve. (**B**) The percentages of witnessed VT/VF OHCA and non-VT/VF patients stratified by SACAF scores. (**C**) The percentages of witnessed VT/VF OHCA and non-VT/VF patients stratified by modified CHA_2_DS_2_-VASc scores.

The SACAF score (male Sex, Age category ≤ 65 years old, Cardiovascular diseases, and ECG rhythm of AF) was composed of these independent predictors with equal weight assigned to each risk factor for predicting initial VT/VF rhythm in patients with witnessed OHCA. The performance of the SACAF score and the modified CHA_2_DS_2_-VASc in discriminating VT/VF from non-VT/VF OHCA as evaluated by AUC was 0.739 (95% CI = 0.681–0.797, *P* < 0.001) and 0.474 (95% CI = 0.408–0.541, *P* = 0.464), respectively (Fig. 3). Using a SACAF cutoff score of ≥ 2 to discriminate VT/VF from non-VT/VF OHCAs yielded a sensitivity of 0.75, a specificity of 0.60, a positive predictive value of 0.29, and a negative predictive value of 0.92.

## Discussion

The principal findings of the THREATEN study included 1) identifying independent risk factors for bystander witnessed VT/VF OHCA in a multicenter cohort based on the in- and pre-hospital data; 2) demonstrating the usefulness of a simple SACAF score for discriminating VT/VF from non-VT/VF OHCAs. These findings may help develop preventive measures to improve survival in these patients.

### AF as an independent risk factor for VT/VF OHCA

SCD has been shown to be an important cause of death in AF patients by analyzing the data from the Atherosclerosis Risk in Communities (ARIC) and Cardiovascular Health Study (CHS) cohorts and from the RE-LY trial patients^27, 28^. Since SCD in the general population is primarily caused by VT/VF in most patients^29^, it raises an important question whether AF and VF are associated and what are the mechanisms linking the two arrhythmias together. Because the diagnosis of SCD was based on clinical information with no ECG documentation of VF in the aforementioned studies, the relationship between AF and VF remains to be defined. Our study revealed that the ECG rhythm of AF adjacent to the OHCA event was an independent predictor for witnessed VT/VF SCD documented by ECG from AED. This finding concurs with the observation by Bardai et al.^14^ in a community-based case–control study, where AF (15.4% of total VF cases) was independently associated with a threefold increased risk of ECG documented VF OHCA compared with age-/sex-matched non-VF subjects from the community. Similarly, in our study, the prevalence of AF in witnessed VT/VF OHCA was 20.1% (17/81), which was associated with a 2.36-fold higher risk for VT/VF compared with controls in this witnessed OHCA population. In a nationwide cohort study, Chao et al. analyzed 352,656 AF and 352,656 non-AF patients without antecedent VT/VF SCD and found that the annual risk of VT/VF SCD was higher in AF than in non-AF groups with an adjusted hazard ratio of 1.64^13^. In patients with ICD implantation, the prevalence of AF was common, and AF was an independent predictor of appropriate ICD therapy^30–32^. All these lines of evidence link AF with a higher risk for VT/VF SCD in the general population and in diseased patients with prior ICD implantation.

The potential mechanisms underlying AF-related higher risk for VT/VF are multifactorial through direct and/or indirect effects or an epiphenomenon^33–35^. These proposed mechanisms involve (1) electrophysiological effects of irregular ventricular rates during AF exposing the ventricle to short-long-short R-R interval sequences and shortened ventricular refractoriness during rapid heart rates in AF, (2) unfavorable hemodynamic consequences of AF-related reduction of cardiac output secondary to the loss of atrial mechanical function and decreased diastolic filling time with a reflex increase in sympathetic tone, and (3) mutations in ion channel–encoding genes associated with both AF and VF. While all these hypotheses provide plausible mechanistic insights toward AF begetting VT/VF, it is imperative to carry out more studies to examine these hypotheses.

### A simple SACAF score in predicting VT/VF OHCA

The current study identified male sex and age ≤ 65 years old as independent risk factors for presenting VT/VF rhythm in witnessed OHCA patients after adjusting for comorbidities and ECG parameters. The association of the two striking demographic factors, younger age and male sex, and the high susceptibility of VT/VF SCD has been replicated in a number of studies in community-based populations and in patients with coronary artery disease or heart failure. We previously showed that in 1629 patients with presumed cardiogenic OHCA, both male sex (adjusted odds ratio 2.45) and age < 65 years (adjusted odds ratio 2.39) were associated with a higher risk for VT/VF OHCA in the THUNDER study^12^. A large-scale nationwide cohort study provided epidemiological data regarding the risk of VT/VF SCD among Asian AF patients with data for each age strata^13^. The AF-associated increase in the risk of VT/VF SCD was higher for patients aged < 75 years than for those aged ≥ 75 years^13^. Among 10,334 patients with acute myocardial infarction, 358 (3.5%) of them experienced life-threatening VT/VF in whom the mean age was younger (61 vs. 63 years old), and there was a male predominance (85% vs. 75%) compared with the non-VT/VF patients^36^. Aktas et al.^37^ compared the risk of the first VT/VF event and the risk of first appropriate ICD shock between younger (< 75 years) and older (≥ 75 years) patients enrolled in the MADIT-CRT trial. The data showed that older patients experienced a significantly lower risk of VT/VF and appropriate ICD shocks than younger patients. All these lines of evidence support the notion that aging and female sex may be associated with a lower incidence of VT/VF, particularly in patients with coronary artery disease.

Given that male sex, age ≤ 65 years old, cardiovascular disease, and AF were identified as independent risk factors for VT/VF OHCA in the current study, we therefore constructed a simple SACAF score tool by assigning equal weight to each of the four risk factors. A SACAF cutoff score of 2 was useful in discriminating VT/VF from non-VT/VF OHCAs with a sensitivity of 0.75, a specificity of 0.60, and a high negative predictive value of 0.92. To validate the score in a separate cohort, a SACAF score of 2 accurately predicted VT/VF in 38 out of 42 patients (90.5%) with ST-elevation myocardial infarction who presented with witnessed VT/VF OHCA (data not shown). To further validate the SACAF score, we also analyzed the performance of a modified CHA_2_DS_2_-VASc score in the study cohort. In MADIT-CRT trial, a low CHA_2_DS_2_-VASc score was associated with a higher risk of ventricular arrhythmias, which was mainly driven by younger age and male sex in heart failure patients^22^. Since the relative weights for age and sex were in the opposite direction between SACAF and CHA_2_DS_2_-VASc, the performance of the SACAF in our patients actually parallels the findings from the MADIT-CRT regarding age and gender effects on VT/VF^22^. In the current study, the modified CHA_2_DS_2_-VASc score was not associated with VT/VF OHCA in our patients, which can be attributed to several factors, such as different patient populations (general vs. heart failure population), insufficient sample size, and coexistence of positive and negative driving factors in the modified CHA_2_DS_2_-VASc score. It should also be noted that the original CHA_2_DS_2_-VASc score was created to estimate stroke risk, and it is expected not to provide clinical value in identifying high-risk patients susceptible to VT/VF OHCA. Furthermore, in recent years, the use of the CHA_2_DS_2_-VASc score in predicting ischemic stroke and thromboembolism has extended beyond the originally proposed AF field^38^. Indeed, further large-scaled studies are needed to confirm whether a high CHA_2_DS_2_-VASc score was inversely associated with presenting VT/VF events in bystander witnessed OHCA patients.

## Limitations

There are several limitations in the current study. First, although the SACAF score appears to be able to discriminate VT/VF from non-VT/VF OHCA, the positive predictive value was relatively low. The lower positive predictive value can be attributable to a higher rate of false positive cases when employing a SACAF cutoff score of ≥ 2 to predict witnessed VT/VF OHCA. Theoretically, incorporation of other risk factors such as left ventricular ejection fraction, severity of coronary artery disease, and myocardial infarction should reduce the false positive rate and improve the positive predictive value. However, from the epidemiological standpoint, we believe that the SACAF score is sufficient to serve as a simple screening tool for VT/VF OHCA in general population with a high negative predictive value and a low false negative rate. Second, a 12-lead ECG record closest to the OHCA event can only be identified in approximately half of witnessed OHCA patients, which may lead to a selection bias in this retrospective-based study. Third, the current study only enrolled witnessed OHCA patients to develop the predictive model, and whether this predictive model can be extended to predict VT/VF OHCA in other population is still unknown. Lastly, inherited genetic defects can cause fatal ventricular arrhythmias, and the current study did not incorporate genetic factors for analysis. Indeed, further large-scale studies with enrollment of more patients and incorporation of more risk factors are needed to improve the risk score.

## Conclusions

The simple SACAF score appears to be useful in identifying high-risk patients susceptible for VT/VF OHCA in comparison with the modified CHA_2_DS_2_-VASc score. These findings also pave the way for future mechanistic evaluation to develop more effective strategies to improve survival in these patients.
